# Partner Relationship Quality and Inclusion of Intimate Partners as Network Confidants of Older Adults

**DOI:** 10.1093/geroni/igaf122.838

**Published:** 2025-12-31

**Authors:** Yiang Li, Linda Waite

**Affiliations:** The University of Chicago, Chicago, Illinois, United States; The University of Chicago, Chicago, Illinois, United States

## Abstract

Robust social networks and high-quality intimate partnerships each promote health in later life, but little is known about how older adults integrate spouses and partners into their core discussion networks. Using data from the National Social Life, Health, and Aging Project (NSHAP), this paper examines the likelihood that older adults name their spouse or partner as a “core confidant” and how relationship quality shapes that designation. Among 1,844 respondents with an intimate partner, nearly one in four did not list that partner as a confidant. Naming a spouse as a confidant is strongly predicted by positive indicators of relationship quality, including time spent together, partner support, and relationship satisfaction. Conversely, partner strain lowers the probability of inclusion. The link between relationship support and confidant naming is most pronounced for highly educated respondents. We discuss the findings as reflecting social inequalities in the ability to integrate intimate ties into broader networks.

